# The Effect of Body-Related Stimuli on Mental Rotation in Children, Young and Elderly Adults

**DOI:** 10.1038/s41598-018-37729-7

**Published:** 2019-02-04

**Authors:** Tina Iachini, Gennaro Ruggiero, Angela Bartolo, Mariachiara Rapuano, Francesco Ruotolo

**Affiliations:** 1 Laboratory of Cognitive Science and Immersive Virtual Reality, Department of Psychology, University of Campania “Luigi Vanvitelli”, Caserta, Italy; 2 University of Lille, CNRS, CHU Lille, UMR 9193 - SCALab - Sciences Cognitives et Sciences Affectives, 59000 Lille, France; 3 Institut Universitaire de France, Paris, France

## Abstract

This study aimed to explore the development of mental rotation ability throughout life by comparing different kinds of stimuli. Thirty-six children (6–9 years-old), 30 young (20–28 years-old) and 30 elderly people (60–82 years-old) performed mental rotation tasks with abstract (i.e. two-dimensional lines) and concrete stimuli (i.e. hands, human/animal faces). The results showed that overall young people outperformed children and elderly people, while children were less accurate than the elderly. However, the effect of age was shaped by the kinds of stimuli: (a) young people were more accurate than children and elderly people particularly with abstract stimuli; (b) elderly people improved their performance with images depicting faces; (c) children performed better with body-related stimuli than animal faces. Finally, performance was more difficult when stimuli were rotated by 180°, especially for younger and older females. We may conclude that the effects of age are modulated by the characteristics of the stimuli with a specific difficulty for abstract stimuli and a facilitation for concrete stimuli. As an innovative aspect, during childhood there appeared a specific facilitation for body-related stimuli, not just for concrete ones. These findings are interpreted according to embodied models of cognitive development and the effects of ageing on the brain.

## Introduction

“*If you can’t imagine things, how can you learn?*”. With this provocative title, The Guardian^1^ recently brought to the attention of the general audience one of the most important human abilities, mental imagery, stressing its fundamental role in learning processes and in most human activities throughout life. If you recall past events and care about the future, visualize a character in a book, or give directions to your house, you are using imagery!

Mental imagery can be defined as the mental simulation of an event, object or scene in the absence of the related perceptual input^2,3^. This simulation is based on the voluntary and controlled reactivation of memorized sensorimotor experiences^4^. Moreover, mental images can be transformed in various ways resulting in more abstract or more concrete mental representations^5,6^. Mental images, then, can be multimodal and dynamic, more or less schematic, and can involve several transformation processes^4,5,7–9^.

Among the possible operations we can do with mental images, mental rotation has been one of the most studied. Through this process, a mental image is rotated along a continuous trajectory in the mental space until it reaches a new orientation^10^. Since the early 70’s, behavioural experiments have shown that when similar objects with different orientations have to be compared, time for making a judgment increases in a near-linear way with the angular disparity between the objects^9,11,12^. This effect has been observed with different kinds of stimuli: from geometric and “abstract” such as letters, lines, polygons and three-dimensional cubes, to embodied and concrete such as hands, legs, and whole-body figures^13–16^.

## Kinds of Rotational Stimuli

Available empirical evidence suggests that mental rotation may rely on two complementary but neurally dissociable imagery strategies depending on the kind of stimuli: a visual-spatial strategy centered on the object and a motor strategy centred on the body^6,9,17^. For example, Parkinsonian patients with reduced motor capacity showed more deficits during the mental rotation of body parts than the mental rotation of letters or three-dimensional objects^18,19^. Within an embodied cognition approach, recent research explored whether stimuli that trigger an action (such as human bodies) could promote the use of sensorimotor embodied strategies and facilitate task performance. Amorim, Isableu and Jarraya^20^ provided abstract three-dimensional Shepard and Metzler’s^10^ shapes with body characteristics by adding a head to the shapes of cubes to evoke a posture. Object recognition was easier with the embodied than abstract stimuli. The facilitation with human bodies as compared to cube figures is a typical result supporting the embodied cognition view^21,22^. However, no study has compared different “living” stimuli such as animals and humans. This point is critical since infants are sensitive to the animated/unanimated distinction even at few weeks of life^23–25^. It is therefore worth testing the possibility that the body-related stimuli facilitation might simply reflect a vantage for living beings.

## Mental Rotation Changes in Lifetime

Several studies have shown that our ability to transform mental images declines with ageing^26^. Very young children of around 5 years of age are able to mentally rotate stimuli^27^, but this ability reaches higher levels during adolescence^28^ and declines with ageing^26,29–36^. For example, Dror and Kosslyn^26^ studied the effects of age on four components of mental imagery: generation, maintenance, inspection and transformation. The authors found that the ability to generate and rotate mental images becomes more difficult with age. Elderly participants found it increasingly harder than young participants processing greater rotational angles. However, the severity of the decline in mental rotation performance often depends on the kind of task, object-based vs. egocentric, and the stimuli used^29,34,37^. In object-based mental rotation tasks, the objects must be judged in relation to each other while the relationship between the environment and the observer remains fixed. In egocentric mental rotation tasks, typically involving body-related stimuli, individuals have to imagine themselves rotating in order to complete the task. Jansen and Kaltner^37^ compared female and male old persons (aged 60–71) on these two mental rotation tasks by using human figures and letters as stimuli. They found a facilitation with human figures and egocentric mental rotation tasks, and observed that males were more accurate than females. The authors concluded that mental rotation performance in older adults is affected by stimulus type, kind of transformation and gender.

As regards gender differences on mental rotation, meta-analysis studies on adults and older children indicate robust effects generally favoring males^38,39^. Although several studies agreed that these differences emerge at the age of around 8 years^40–42^, the question is still matter of debate. While many studies report no differences before puberty^43–45^, others have argued that these differences can be detected when the task is adequately modified^42,46,47^. In other words, it is necessary to devise mental rotation tasks that are at an appropriate level of difficulty for children.

In many studies involving children, the task is adapted either by exchanging geometric figures for human hands and avoiding three-dimensional stimuli, or by measuring success without considering response time^48^. The mental rotation tasks adopted with older children and adults usually use response time as a measure, but this may be not appropriate for younger children because of the attentional demands of speeded responses^47^. For this reason, accuracy has often been used as a reliable measure^27,47,49^. Moreover, prior works showed that tasks involving two-dimensional stimuli are easier than those carried out with three-dimensional stimuli^38,47^. Indeed, in most studies about the development of mental rotation, the three-dimensional forms of the Shepard and Metzler paradigm were replaced by two-dimensional stimuli^50,51^. For all these reasons, we used two-dimensional black and white line drawings on A4 papers and we avoided speeded responses in favor of accuracy. Previous studies on spatial memory during the entire lifetime have shown a better performance in young adults as compared to children and elderly people, with a specific difficulty at 6/7 years of age and at 70/80 years of age^52^. These age ranges are therefore important in order to outline the development of mental rotation ability throughout lifespan.

In sum, it is still unclear how mental rotation ability changes during the entire lifespan and whether the kinds of stimuli to be rotated play a role. So far, no study has directly assessed this issue. We know that children develop first a concrete thinking focused on the physical world and then on abstract elements^53^. Moreover, previous evidence with older adults suggests a facilitation with body-related, concrete and familiar stimuli^34,37,54^. Therefore, it is possible that age-related changes in mental rotation ability interact with the capacity of individuals to process more concrete or abstract stimuli. For this reason, we explored the influence of different types of stimuli on mental rotation ability during the developmental course (from childhood to elderly age) by using classic tasks (mental rotation of abstract lines and hands) and devising a new task (the Ears task, comparing animal and human faces).

Children (6–9 years old), young (20–28 years old) and elderly people (60–82 years old) were required to perform mental rotation tasks with “abstract” (i.e. two-dimensional lines) and “concrete” stimuli (i.e. hands, human and animal faces; for an example see below “Materials and tasks”). These stimuli could be rotated by 90, 180, and 270 degrees with respect to their upright position (i.e. 0°). We hypothesized an effect of age with young people performing better than children and elderly people. We also hypothesized an effect due to the “concreteness” of the stimuli, such that mental rotation should be more accurate with concrete (i.e. hands, animal and human faces) than abstract stimuli (i.e. abstract lines). The negative effect of age should presumably be stronger with abstract lines. According to previous evidence about embodied stimuli^20,22^, it is also possible to hypothesize a facilitation for human than animal stimuli. Finally, we checked the data for gender differences.

## Results

### Comparison between the four categories of Stimuli x Age

**Figure 1:**
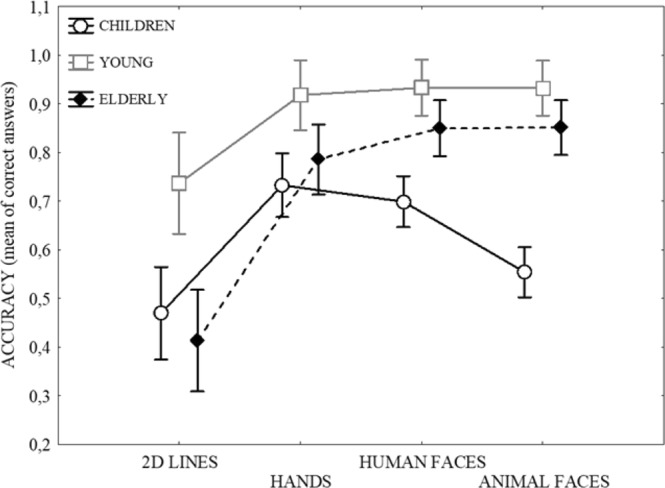
Mean accuracy in the four mental rotation tasks as a function of Age Groups. Vertical bars denote 0.95 confidence intervals.

The results revealed a main effect of Age on mean rotational accuracy: F(2, 93) = 34.528, p = 0.000001, *ƞ*^2^_*p*_ = 0.43. The related means were: Young = 0.880, SD = 0.173; Old = 0.725, SD = 0.166; Children = 0.614, SD = 0.234. The post-hoc test showed that young adults were more accurate than elderly participants and children (at least p < 0.001), and children were less accurate than the other groups. A main effect of Stimuli also emerged: F(3, 93) = 49.789, p = 0.000001, *ƞ*^2^_*p*_ = 0.35. The related means were: Lines = 0.535, SD = 0.316; Hands = 0.807; SD = 0.211; Human faces = 0.819, SD = 0.185; Animal faces = 0.765, SD = 0.227. The two-dimensional lines were less accurate than all other stimuli (at least p < 0.0001). These effects were qualified by a significant interaction: F(6, 279) = 6.515, p = 0.00001, *ƞ*^2^_*p*_ = 0.12 (see Fig. 1). The post-hoc analysis showed that in both young and elderly adults mental rotation was worse with abstract L-shaped lines than all other concrete stimuli (at least p < 0.01), whereas the three categories of concrete stimuli did not significantly differ. Instead, in children the performance was worse with abstract L-shaped lines than human hands/faces (at least p < 0.0001). Bodily stimuli, i.e. hands and faces, were more accurate than animal faces (at least p < 0.05), while two-dimensional lines and animal faces did not differ. The comparison between groups showed that young participants performed better than both children and elderly individuals with abstract L-shaped lines (at least p < 0.0001). With all concrete stimuli, young participants performed better than children (at least p < 0.01) but not elderly participants. Children showed a specific difficulty with animal faces: they performed worse than young and elderly people (at least p < 0.0001).

We tested the probability of guessing the correct responses by chance (that is higher in double-choice than four-choice tasks) by using the binomial analysis. The mean binomial probability of guessing the correct responses for each task was the following: Lines = 28.062%, Hands = 14.898%, Human faces = 11.199%, Animal faces = 17.586%. These results clearly indicate that the probability that our participants guessed the correct responses by chance was higher for the Lines task than for the others, thus ruling out the possibility that the number of response alternatives could explain the pattern of results obtained.

Finally, we corrected the participants’ scores by using the formula reported in the Method section. The ANOVA on mean corrected scores confirmed the previous effects: a main effect of Age (F(2, 93) = 37.734, p = 0.000001, *ƞ*^2^_*p*_ = 0.45), a main effect of Stimuli (F(3, 93) = 13.911, p = 0.000001, *ƞ*^2^_*p*_ = 0.13, an Age by Stimuli interaction: F(6, 279) = 7.429, p = 0.00001, *ƞ*^2^_*p*_ = 0.14. The mean actual and corrected scores had the same standardized alpha = 0.70 and average inter-item correlation = 0.38. Cronbach’s alphas are all adequate: corrected scores = 0.69, actual scores = 0.66. This indicates that the two scores are equally reliable. These analyses gave us confidence in the robustness of the data and for this reason we left the original mean scores for the separate analyses on the single tasks.

## Separate ANOVAs

### Lines

A main effect of Age emerged, (F(2, 93) = 10.983, p = 0.0001, *ƞ*^2^_*p*_ = 0.19). The mean accuracy of the three groups was: Young = 0.737, SD = 0.325; Old = 0.413, SD = 0.273; Children = 0.469, SD = 0.266. Young participants performed better than both children and elderly people (at least p < 0.001), whereas no significant difference between children and elderly participants was observed (p = 0.711). With the factor “Gender”, neither main effect nor interaction with Age emerged.

### Hands

**Figure 2:**
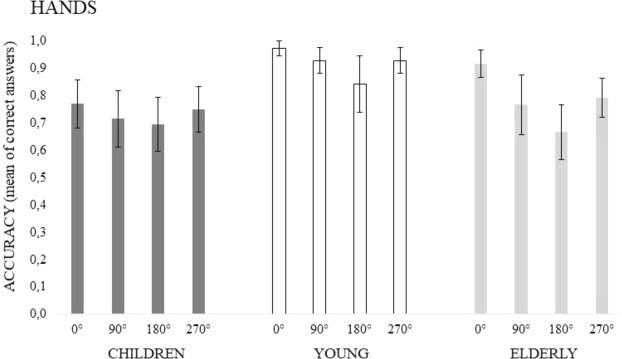
Mean accuracy in the Hand task as a function of Rotational Angles and Age Groups. Vertical bars denote 0.95 confidence intervals.

The ANOVA showed main effects of Age, (F(2, 93) = 7.398, p = 0.001, *ƞ*^2^_*p*_ = 0.14) and Angles of rotation (F(3, 279) = 14.471, p < 0.00001, *ƞ*^2^_*p*_ = 0.13), but no interaction between the two factors (F(3, 279) = 1.795, p = 0.101). As regards Age, the related means were: Young = 0.918, SD = 0.124; Old = 0.785, SD = 0.190, and Children = 0.733, SD = 0.249. The post-hoc test confirmed that young participants performed better than children and elderly participants (at least p < 0.05), whereas no significant difference between the latter appeared (p = 0.531). As regards Angles of rotation, performance was more accurate when stimuli were not rotated (0°) than all other conditions (at least p < 0.05). At the same time, performance was less accurate when stimuli were rotated by 180° than all other conditions (at least p < 0.05). No significant difference between 90° and 270° appeared (p = 0.804). The pattern of accuracy as a function of both Age and Angles is shown in Fig. 2. When checking for Gender effects, a 3-way (Age X Angles X Gender) interaction emerged (F(6, 270) = 2.422, p < 0.05, *ƞ*^2^_*p*_ = 0.05). Performance was less accurate with 180° hand rotation by elderly females than with all angular degrees by young people (at least p < 0.05). A similar effect appeared with female children who were less accurate than both male-female young adults at 0° (p = 0.05).

### Human faces

**Figure 3:**
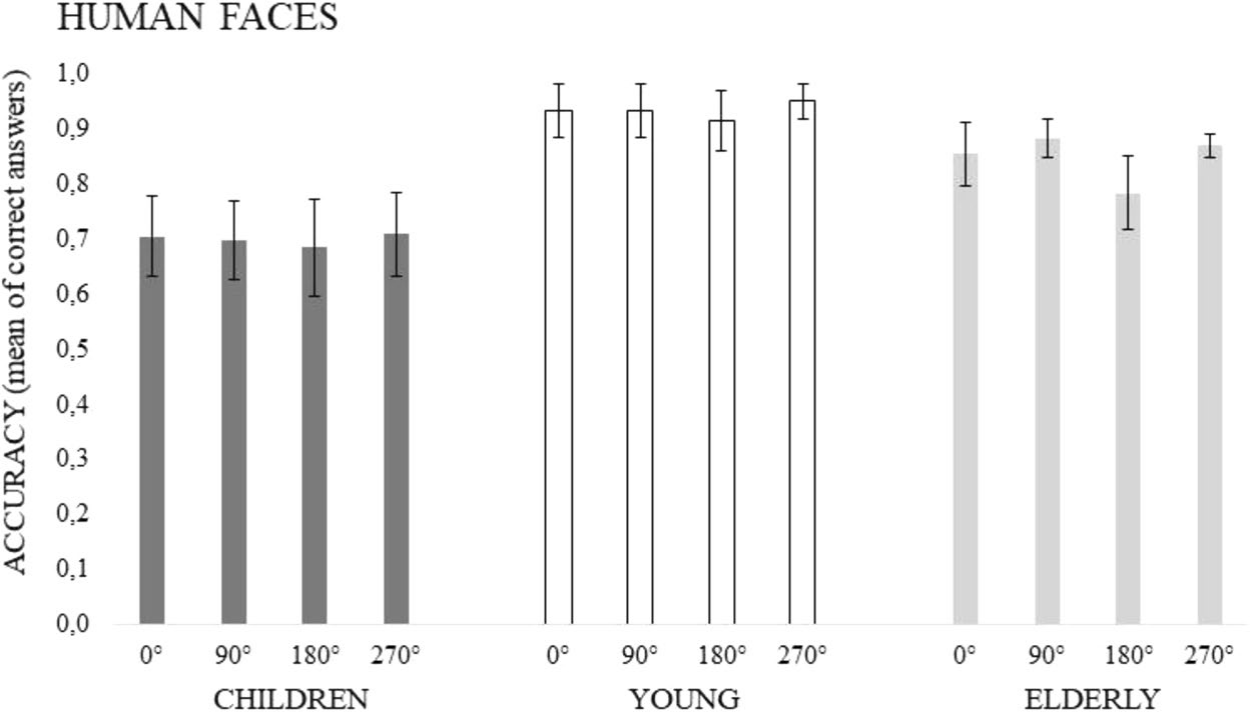
Mean accuracy in the Human faces task as a function of Rotational Angles and Age Groups. Vertical bars denote 0.95 confidence intervals.

The results revealed main effects of Age (F(2, 93) = 18.677, p = 0.00001, *ƞ*^2^_*p*_ = 0.29) and Angles of rotation (F(3, 279) = 4.757, p = 0.005, *ƞ*^2^_*p*_ = 0.05), but no interaction (F(3, 279) = 1.297, p = 0.258). As regards Age, the related means were: Young = 0.933, SD = 0.116; Old = 0.848, SD = 0.089, and Children = 0.699, SD = 0.221. Children performed worse than both Young and Old participants (p < 0.01). Young people were more accurate than children (p < 0.01) but not old people (although p = 0.097). As regards Angles, mental rotation was less accurate when stimuli were rotated by 180° compared to the remaining angles (p < 0.05). No further significance emerged. Neither main effect nor interaction due to Gender appeared. The effect of Age and Angles factors is shown in Fig. 3.

The “Front/Back” x “Age” ANOVA showed a “Front/Back” main effect (F(1, 93) = 14.375, p = 0.0003, *ƞ*^2^_*p*_ = 0.13) but no interaction with the Age factor (F < 1). Performance was more accurate in Back (M = 0.875, SD = 0.193) than Front (M = 0.762, SD = 0.267) perspective.

### Animal faces

**Figure 4:**
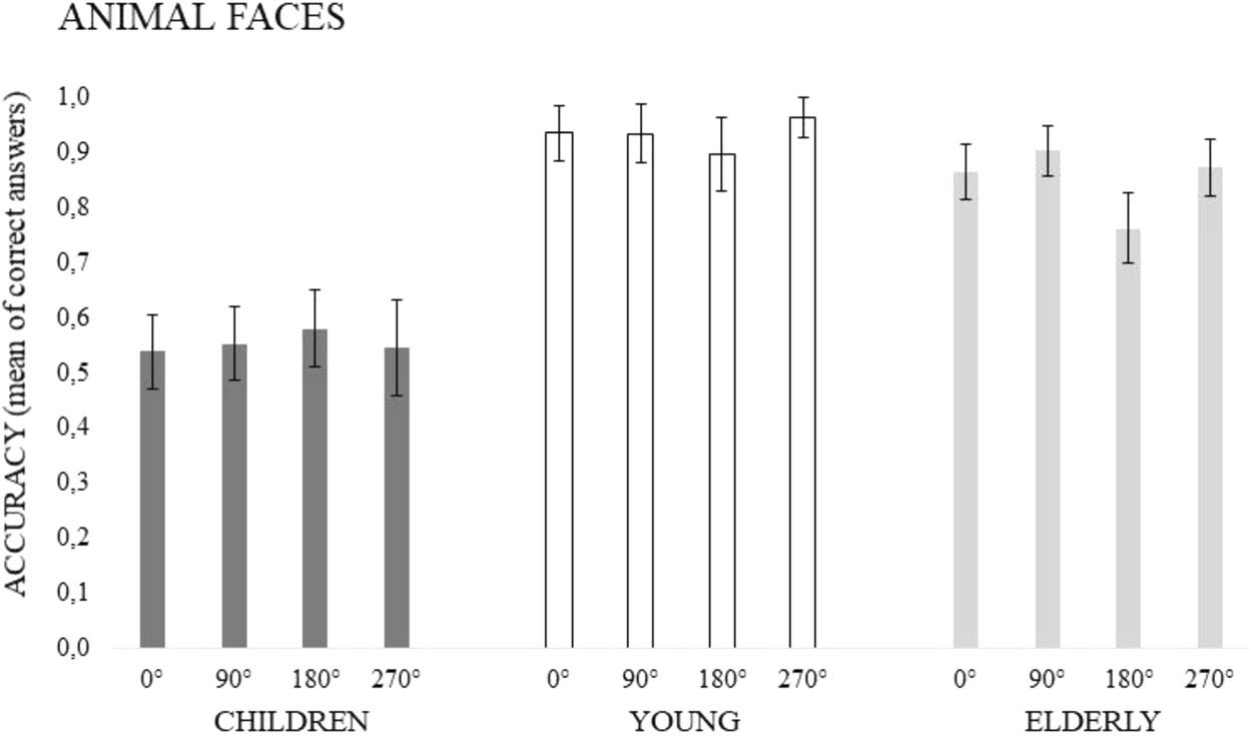
The figure shows mean accuracy in the Animal faces task as a function of Rotational Angles and Age Groups. Vertical bars denote 0.95 confidence intervals.

The ANOVA showed main effects of Age (F(2, 93) = 55.015, p = 0.00001, *ƞ*^2^_*p*_ = 0.54) and Angles of rotation (F(3, 279) = 4.486, p = 0.005, *ƞ*^2^_*p*_ = 0.05). As regards Age, the related means were: Young = 0.932, SD = 0.130; Old = 0.851, SD = 0.111, and Children = 0.554, SD = 0.201. The post-hoc analysis confirmed that children performed worse than both young and old participants (at least p < 0.0001), while Young and Old participants did not significantly differ (p = 0.112). As regards Angles, mental rotation was less accurate when stimuli were rotated by 180° than 90° and 270° (p < 0.05). A significant interaction also emerged (F(6, 279) = 4.682, p < 0.0005, *ƞ*^2^_*p*_ = 0.09). The post-hoc test showed that Children performed worse than Young and Old adults at all angular degrees (at least p < 0.005), while no significant difference between Young and Old adults appeared. Old participants performed worse at 180° than all the other degrees (at least p < 0.01). Neither main effect nor interaction due to Gender appeared. The effect of Age and Angles factors is shown in Fig. 4.

The “Front/Back” x “Age” ANOVA showed a “Front/Back” main effect (F(1, 93) = 4.001, p = 0.05, *ƞ*^2^_*p*_ = 0.04) and no interaction with the Age factor (F < 1). Performance was more accurate in Back (M = 0.800, SD = 0.274) than Front (M = 0.730, SD = 0.297) perspective.

## Discussion

This study aimed to explore how mental rotation ability changes throughout life and verify if the changes were affected by the characteristics of stimuli. To this aim children, young and elderly people had to mentally rotate abstract (i.e. two-dimensional lines) and concrete stimuli (i.e. hands, human and animal faces). In line with our hypotheses, results showed that both age and types of stimuli affect rotational ability. A general difficulty in children and elderly people as compared to young people emerged. More specifically, young people performed better than children and elderly people, while children were less accurate than the elderly. As regards the types of stimuli, the overall performance with the abstract L-shaped lines was less accurate than all other stimuli, thereby confirming a general advantage for concrete stimuli^54^. Moreover, the better mental rotation performance of the young people compared to children and elderly people was particularly evident with abstract stimuli. As regards concrete stimuli, young people performed better than children with all the stimuli, but better than elderly people only with hands stimuli. Both young and elderly individuals performed worse with abstract stimuli than with the three types of concrete stimuli for which, instead, they showed a similar performance. Moreover, all age groups performed better when human and animal faces were shown in the back perspective than the front one. This suggests that at all ages a cost is paid to assume the perspective of another person, as it is typical of egocentric mental rotation transformations^37^. This pattern of findings supports the hypothesis that the negative impact of age on mental rotation is stronger with abstract stimuli. Instead, the processing of concrete and familiar stimuli such as animal and human faces seems to be more resistant to the effect of ageing, thus facilitating the performance of older people^34,54^. Finally, performance was overall more difficult when stimuli were rotated by 180°. This angle represented the contra-aligned orientation with higher angular disparity from the upright 0° position (90°–270° represent similar right-left axes). The 180° rotation was particularly affected by age and gender effects. Indeed, the processing of 180° rotated hands was harder for younger (i.e. children) and older females than young adults. Furthermore, with animal faces, elderly adults performed worse at 180° than all other degrees.

Mental rotation relies on the capacity to manipulate visuo-spatial information and therefore is a highly active spatial task that requires important attentional resources of the central executive^55,56^. It is possible that the difficulty shown by children and elderly people would reflect the process of maturation (in childhood) and deterioration (in aging) of the frontal cerebral areas supporting executive functions. It is well known that normal aging is characterized by reductions in volume of the frontal cortex that correlates with a general decline in executive and attentional functions^57,58^. A similar difficulty in childhood would be due to their frontal lobes still under development^59–63^. Brain related changes in lifetime could explain why young adults overall outperformed children and old people.

The difficulty at all ages with abstract L-shaped lines compared to concrete stimuli confirms that mental images can be more easily manipulated throughout lifespan when they represent living beings. This is consistent with previous literature demonstrating a facilitation with body-related stimuli, especially in elderly individuals^20,37^. However, the overall pattern showed some unexpected findings in children. Even though children behaved worse than young people with all the stimuli, they showed a specific difficulty, even compared to the elderly, with the animal faces. Separate analyses confirmed that children performed worse than young and old adults with animal faces. Children found it more difficult to process abstract lines and faces of animals than human hands and faces. In other words, they were facilitated with stimuli representing human body-parts compared to animal faces and abstract lines, with no difference between the latter two. The effect cannot be ascribed to concreteness per se or sensitivity to animated/unanimated categorization^23–25^, since no facilitation for animal faces appeared. It could reflect a specific advantage for the information related to the human body.

Overall, the pattern of results is consistent with the idea that mental rotation may engage a visuo-spatial object-centered strategy or a motor body-centered strategy^6,9,17,37^. Mental rotation of body parts should involve motor imagery, whereas mental rotation of L-shaped lines visuo-spatial imagery^16,18,19,64^. Motor imagery can be defined as an internal simulation of movements from a first-person perspective without any overt physical movement^65–70^. Instead, visuo-spatial imagery involves the representation of the spatial components of the stimulus with alleged pictorial features^3,64,71^.

How can we explain the body-vantage in childhood? Within the literature on embodied cognition, the motor and body-based nature of a series of cognitive processes is highlighted^72–74^. The sensorimotor system may serve to represent abstract ideas^75,76^ and can even facilitate object recognition through mental rotation by providing abstract stimuli with bodily features^10,20^. Behavioral^11,77–79^ and neuroscientific evidence^3,79^ suggests that mental imagery and sensory-motor systems share common mechanisms and common neural areas. In particular, when mental rotation involves body parts (e.g. hands), an activation of motor areas is reported^6,12,80,81^. Moreover, neuroimaging evidence has shown that mental imagery of hand, foot and tongue movements activates somatotopical sections of the human motor cortices^82^. Motor imagery, then, engages in a systematic way the somatotopically organized sections of the motor cortex^83^. If we assume that mental rotation of body-part stimuli engages motor imagery, then we should conclude that somatotopic motor areas are also activated. These patterns of activation should support various forms of mental simulations that are embodied not only because occur at neural level but also because they represent motor properties relying on a pre-existing body-model in the brain, and therefore involve a non-propositional form of self-representation^84^. A similar mechanism should be “innate” and not learned with experience, so already available in childhood. Cognitive processes exploiting this mechanism should therefore be facilitated.

Before concluding, it is important to make some considerations about the effect of gender. Much research has shown that men typically outperform women in mental rotation tasks^38,39,85^. Instead, our results did not show a general female difficulty. Voyer and Jansen^22^ resumed several possible social and cognitive explanations for gender differences. Among the cognitive factors, they mentioned time pressure, task complexity or stimulus type. Indeed, the literature shows that gender differences in mental rotation are larger when the task is administered with time constraints rather than without^86^. In addition, many studies with adult participants showed sex differences with both two-dimensional and three-dimensional mental rotation tasks^11^ but the effect is generally stronger with three-dimensional stimuli^38^. Therefore, it is likely that the absence of general gender differences in our study can be ascribed to the lack of time constraints and the adoption of two-dimensional stimuli.

A gender difference only emerged with children and old females when stimuli were rotated by 180°. This angular disparity requires a longer and more demanding rotation process than other angles within the range considered here and, presumably, should involve the capacity to process the stimulus as a whole by adopting an object-based allocentric strategy (for which a female difficulty is often reported^38,87,88^. These factors could explain the difficulty faced by women in childhood and old age. However, why this effect was only revealed with stimuli depicting hands is trickier to explain and further studies would be needed.

In conclusion, the current study shows that mental rotation ability changes throughout lifetime and differences between children, young and elderly people are modulated by the kinds of stimuli. Indeed, a specific difficulty with abstract stimuli, especially in childhood and elderly age, and a facilitation with concrete stimuli emerged. Importantly, children were facilitated with body-related stimuli compared to animal faces and abstract lines. From an embodied cognition perspective, this might reflect a vantage due to reliance on an innate model of the human body in our brain. The overall findings suggest that the mental rotation ability is primarily rooted in embodiment processes and that efficient spatial transformations of more abstract images are possible when the development of the brain has reached its full maturation.

## Methods

### Participants

The sample comprised three different age groups: 36 children (aged 6–9 years, mean age = 7.50, SD = 1.21), 30 young (aged 20–28 years, mean age = 22.13, SD = 2,16) and 30 old (aged 60–82 years, mean age = 67.17, SD = 7.29) individuals. Mean education (in years) of the three groups was: children = 2.5, young = 16.1, old = 13.96. As regards children, they were recruited from a public elementary school of Naples (Italy), with the consent of the education authorities. The experimental protocol was administered in the presence of the teachers in a calm room of the school. The following inclusion criteria were met for each child: age range from 6 to 9 years, typical development, absence of psychological or neurological disorders reported by teachers or parents. All children were invited to get at ease and to take as long time as they thought necessary to communicate their response during testing. As regards young adults, they were recruited from a panel of Psychology students (Department of Psychology, University of Campania “L. Vanvitelli”) who volunteered to participate in the experiments as practical experience during their university courses. They were tested in the Laboratory of Cognitive Science & Immersive Virtual Reality (CSIVR) of the Department of Psychology (University of Campania). As regards old adults, they were recruited from general practitioners of several cities of the Campania region (Italy) or were relatives/friends of Psychology students. They were tested in the CSIVR Lab (in the few cases where this was not possible, a quiet room was chosen in private homes). The number of males and females within each age group was balanced. Young and elderly participants were submitted to a test of general cognitive abilities, the Mini Mental State Examination (MMSE)^89,90^. No one reported a score below 28 (cutoff = 23.8). All participants were free from neurological and psychiatric disorders as reported by their medical history. All participants had normal or corrected-to-normal vision. They were explicitly asked to report possible perceptual deficits (for minors, their parents were asked). Each participant was tested individually in a single session lasting about 20 minutes. The experimenters who ran the experiment and all participants were blind to the hypotheses of the study.

### Ethical approval and informed consent

All adult participants provided written informed consent to participate in the study. For children participants, written informed consent for study participation was provided by their parents. Moreover, verbal consent from each child was also obtained. Recruitment and testing were carried out in accordance with the relevant guidelines and regulations of the Declaration of Helsinki (World Medical Association, 2013). All experimental protocols were approved by the local Ethics Committee of the Department of Psychology (prot. #151549), University of Campania Luigi Vanvitelli (Caserta, Italy).

### Materials and tasks

**Figure 5:**
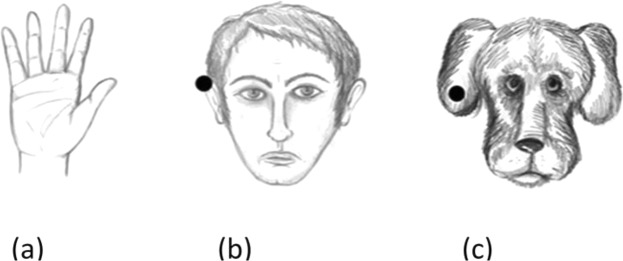
The figure depicts an example of the stimuli used in the mental rotation tasks: (**a**) mental rotation of hands; (**b**) mental rotation of human faces; (**c**) mental rotation of animal faces.

Stimuli represented four different categories: abstract two-dimensional lines, human hands, human faces and animal faces (see Fig. 5). All the stimuli consisted of black and white line drawings pictures printed on A4 sheets of paper. For each task accuracy (1 = correct; 0 = incorrect) was recorded.

#### Mental rotation of lines

The task was drawn from the standardized Battery for Visuo-spatial Abilities (BVA, known in Italy as TERADIC)^91^. Stimuli consisted of ten L-shaped lines with one dot. One target-line appeared on the left side and four lines on the right side of an A4 sheet placed horizontally. Among the four lines only one corresponded to the target-line except that it was rotated clockwise or counterclockwise. Participants had to identify among the three distracters, the line that matched the target-line. Mean accuracy was computed (total score 0–10).

#### Mental rotation of hands

Participants were presented with 32 black and white drawings of hands (the left and the right hand), in back and palm perspective (see Fig. 5a; all concrete stimuli were drawn by T. Iachini). Hands were presented one at a time, according to four different orientations with respect to their 0° upright position: 0°, 90°, 180° and 270°. Participants were asked to provide right/left laterality judgments. More specifically, they were instructed to look at the hand and decide if it was the right or the left hand. For each rotational angle, mean accuracy was computed (total score 0–32, 0–8 by angle).

#### Mental rotation of human faces

Participants were presented with 32 black and white drawings of male and female human faces (see Fig. 5b). The faces were presented one at a time, in front and back perspective, according to four different orientations (0°, 90°, 180°, 270°) with respect to the 0°. On one ear, a black dot appeared. Participants had to decide if the dot was on the right ear or the left ear the human face. For each rotational angle, mean accuracy was computed (total score 0–32, 0–8 by angle).

#### Mental rotation of animal faces

Participants were presented with 32 black and white drawings of cat and dog faces (see Fig. 5c). The faces were presented one at a time, in front and back perspective, according to four different orientations (0°, 90°, 180°, 270°) with respect to the 0°. On one ear, a black dot appeared. Participants had to decide if the dot was on the right ear or the left ear. For each rotational angle, mean accuracy was computed (total score 0–32, 0–8 by angle).

### Procedure

The order of presentation of the four mental rotation tasks was counterbalanced across participants. Each drawing was presented one at a time on the table by the experimenter. Participants were allowed to rest as needed between the tasks. There were some practice trials at the beginning of the session. The experimenter made sure that the procedure was clear and, if all went well, started administering the tasks. At the ending, participants were interviewed to be sure that they had followed the instructions accurately.

### Data analysis

Independent Variables: Age (child, young, elderly) as between factor; Stimuli (Lines, Hands, Human and Animal Faces) and Angles of rotation (0°, 90°, 180°, 270°) as within factors. In all ANOVAs, 3 groups were compared: 36 children (6–9), 30 young (20–18) and 30 old (60–82) adults. All analyses were performed on mean accuracy. The mean accuracy by each participant was computed (wrong = 0, correct = 1). Skewness and Kurtosis distributions of our study variables were examined to determine whether the data were normally distributed. They both fell within acceptable ranges (Skewness from −0.800 to 0.011 and Kurtosis from −1.195 to 0.038) suggesting non-normality is not an issue in the present data^92^. Two series of analyses were planned:

- a 3 (Age) x 4 (Stimuli) ANOVA on mean accuracy of each mental rotation task with the aim of testing the effect of Age on the four categories of rotational stimuli: Lines, Hands, Human and Animal Faces. Since the probability of guessing the right response by chance is higher in double-choice (Hands, Human and Animal Faces) than four-choice (Lines) tasks, we computed the chance probability associated with the observed scores for each subject in each task to obtain the actual binomial distribution of the probability of guessing. It is possible that the parameters of the Line task (low number of items and higher response options) could have made it more difficult than other tasks. Therefore, to increase the validity of the comparison across the four tasks we corrected the related scores for random guessing by adopting the formula scoring^93–95^: R − W/(C − 1), where R = right responses, W = wrong responses, and C = number of response choices. Since this formula reduces the impact of wrong responses with the increase of response choices, the correction penalizes the concrete tasks more than the Line task. After applying the score correction, we re-analyzed the mean corrected scores using the same 3 × 4 ANOVA model;
- separate ANOVAs on each category of stimuli: a one-way ANOVA with the factor Age on Lines; 3 (Age) x 4 (Angles of rotation) ANOVAs on Hands, Human Faces and Animal Faces. We also checked for a possible effect of “Gender” by repeating the ANOVAs with the additional between factor “Gender”. Finally, the Human and Animal Faces were presented in front and back perspectives. To test the possibility that especially younger children could be impaired in the front Faces condition that, differently from back Faces, requires changing their perspective to take that of the stimuli, separate analyses for front and back Faces were carried out. We performed separate ANOVAs on the mean scores of Human and Animal Faces using “Front/Back” as within factor and “Age” as between factor.

The Tukey HSD test was used to analyze post-hoc effects and effect sizes were expressed by *ƞ*^2^_*p*_.

## Data Availability

The datasets generated and analysed during the current study are available on reasonable request to T. Iachini (santa.iachini@unicampania.it).
